# Development of Digital Twin Clinical Decision Support Tool for Alzheimer’s Prediction and Prevention

**DOI:** 10.1093/geroni/igaf122.3178

**Published:** 2025-12-31

**Authors:** Alex Bangs, Thomas Paterson, Jennifer Rohrs, Don Breuner, Cory Funk

**Affiliations:** Fulcrum Neuroscience, Inc., Minnesota, United States; Fulcrum Neuroscience, Inc., Palo Alto, California, United States; Fulcrum Neuroscience, Inc., Palo Alto, California, United States; Fulcrum Neuroscience, Inc., Palo Alto, California, United States; Fulcrum Neuroscience, Inc., Palo Alto, California, United States

## Abstract

The Fulcrum Brain Health and Neurodegeneration (BHN) model is the product of reconciling over 1,500 peer reviewed papers and accompanying data sets. Our model is coupled with a computational software platform to create a simulated population of millions of virtual individuals representing genetic and environmental variations observed in clinical and observational studies. Using a Bayesian approach, data from individuals in a clinic or trial setting are used to create digital twins. Each digital twin can simulate the predicted risk of mild cognitive impairment (MCI) and dementia with age. Additionally, we have performed preliminary assessments of a set of established interventions around lifestyle interventions, off-label drugs and supplements and how they can be personalized to extend normal cognition. Our digital twins can be further evaluated for these combination therapies, to provide a rank-ordered set of intervention combinations that are predicted to be most effective. We’ve developed a user interface for reviewing digital twin results, starting with a static, graphical prototype and then a running prototype able to create digital twins based on user-provided data. We refined these prototypes following interviews with gerontologists, neurologists, neuropsychologists, and primary care physicians. Interviews included discovery questions about how cognitive assessments were done, how treatments were selected, and how the clinical workflow operated, in addition to feedback on the presented prototype. Anonymized patient cases were used to create digital twins and discuss how the results could be utilized. This process resulted in a roadmap for development including integration into electronic health record systems and clinical workflows.

